# 5,7-Dihy­droxy-3,6-dimeth­oxy-2-(4-meth­oxy­phen­yl)-4*H*-chromen-4-one monohydrate

**DOI:** 10.1107/S1600536810039012

**Published:** 2010-10-02

**Authors:** Akhtar Mohammad, Itrat Anis, Vickie McKee, Josef W. A. Frese, Muhammad Raza Shah

**Affiliations:** a H.E.J. Research Institute of Chemistry, International Center for Chemical and Biological Sciences, University of Karachi, Karachi 75270, Pakistan; b Department of Chemistry, University of Karachi, Karachi 75270, Pakistan; c Department of Chemistry, Loughborough University, Leicestershire LE11 3TU, England

## Abstract

The title compound, C_18_H_16_O_7_·H_2_O, is a flavonoid isolated from *Dodonaea viscosa­*. The benzopyran ring system of the flavonoid is essentially planar [maximum deviation = 0.025 (2) Å] and inclined at 5.83 (2)° to the attached benzene ring. The water of hydration is involved in extensive hydrogen bonding, assembling the mol­ecules into a supra­molecular network *via* classical inter­molecular O—H⋯O hydrogen bonding. The crystal structure is further stabilized by π–π stacking inter­actions [centroid–centroid distance between benzene rings = 3.564 (3) Å].

## Related literature

For the anti-oxidant activity of flavonoids, see: Pedrielli *et al.* (2001 ▶, for their anti-protozoal activity, see: Calzada *et al.* (1999 ▶) and for their anti-viral activity, see: Lin *et al.* (1999 ▶). For hydrogen-bond motifs, see: Etter *et al.* (1990 ▶). For related structures, see: Arfan *et al.* (2010 ▶); Azhar ul *et al.* (2004 ▶); Ferheen *et al.* (2005 ▶); Hussain *et al.* (2008 ▶, 2009 ▶); Jan *et al.* (2009 ▶); Khan *et al.* (2005*a* ▶,*b* ▶); Nisar *et al.* (2010 ▶); Riaz *et al.* (2002 ▶); Sharif *et al.* (2005 ▶).
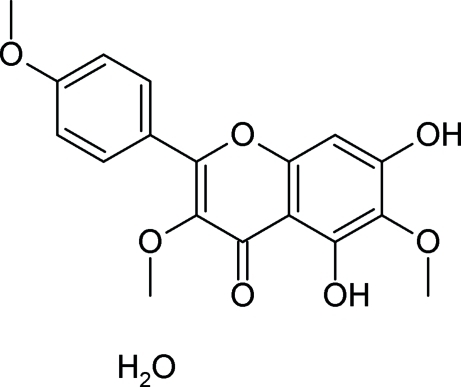


## Experimental

### 

#### Crystal data

C_18_H_16_O_7_·H_2_O
*M* *_r_* = 362.32
Monoclinic, 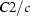

*a* = 19.869 (4) Å
*b* = 6.8126 (15) Å
*c* = 24.424 (5) Å
β = 91.298 (4)°
*V* = 3305.2 (12) Å^3^
*Z* = 8
Mo *K*α radiation
μ = 0.12 mm^−1^
*T* = 150 K
0.19 × 0.18 × 0.09 mm

#### Data collection

Bruker APEXII CCD diffractometer
Absorption correction: multi-scan (*SADABS*; Sheldrick, 2008*a* ▶) *T*_min_ = 0.978, *T*_max_ = 0.990
14127 measured reflections
3400 independent reflections
2072 reflections with *I* > 2σ(*I*)
*R*_int_ = 0.068

#### Refinement

*R*[*F* ^2^ > 2σ(*F* ^2^)] = 0.050
*wR*(*F* ^2^) = 0.142
*S* = 1.05
3400 reflections
243 parameters
3 restraints
H atoms treated by a mixture of independent and constrained refinement
Δρ_max_ = 0.28 e Å^−3^
Δρ_min_ = −0.28 e Å^−3^

### 

Data collection: *APEX2* (Bruker, 2008 ▶); cell refinement: *SAINT* (Bruker, 2008 ▶); data reduction: *SAINT*; program(s) used to solve structure: *SIR2004* (Burla *et al.*, 2005 ▶); program(s) used to refine structure: *SHELXL97* (Sheldrick, 2008*b* ▶); molecular graphics: *SHELXTL* (Sheldrick, 2008*b* ▶); software used to prepare material for publication: *SHELXTL*.

## Supplementary Material

Crystal structure: contains datablocks I, global. DOI: 10.1107/S1600536810039012/rk2231sup1.cif

Structure factors: contains datablocks I. DOI: 10.1107/S1600536810039012/rk2231Isup2.hkl

Additional supplementary materials:  crystallographic information; 3D view; checkCIF report

## Figures and Tables

**Table 1:** Hydrogen-bond geometry (Å, °)

*D*—H⋯*A*	*D*—H	H⋯*A*	*D*⋯*A*	*D*—H⋯*A*
O2—H2*A*⋯O1*W*^i^	0.84	1.92	2.712 (3)	157
O2—H2*A*⋯O3	0.84	2.33	2.780 (3)	114
O4—H4*A*⋯O5	0.84	1.85	2.589 (3)	146
O1*W*—H1*A*⋯O5	0.85 (2)	2.05 (2)	2.886 (3)	165 (3)
O1*W*—H1*A*⋯O6	0.85 (2)	2.71 (3)	3.288 (3)	126 (3)
O1*W*—H1*B*⋯O4^ii^	0.86 (2)	2.38 (2)	3.135 (3)	148 (3)

Symmetry codes: (i) 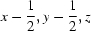; (ii) 
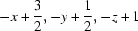
.

**Fig. 1.**
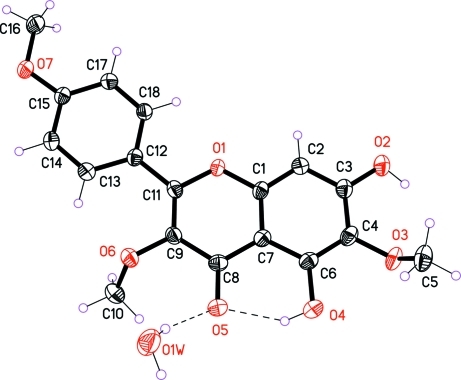
Molecular structure of title compound with atom numbering scheme. Displacement ellipsoids are drawn at 50% probability level. H atoms are presented as a small spheres of arbitrary radius. Several H bonds are drawn by dashed lines.

**Fig. 2.**
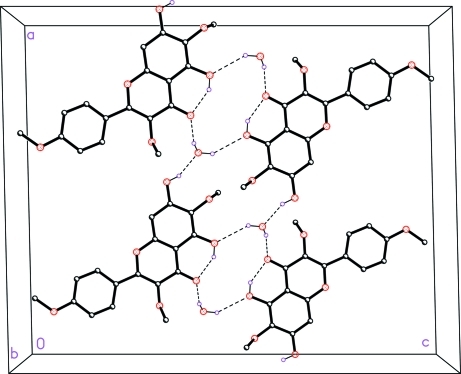
Packing diagram showing the H–bond network (dashed lines).

**Fig. 3.**
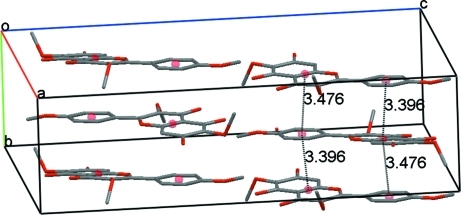
The π–π interactions. Red cirles mark centroids of bonds or rings.

## supplementary crystallographic information

### Comment

Our investigation on natural product chemistry (Arfan *et al.*, (2010); Azhar *et al.*, (2004); Ferheen *et al.*, (2005); Hussain *et al.*, (2008, 2009); Jan *et al.*, 2009) is intended to explore the medicinal aspect of indigenous plants (Khan *et al.*, (2005*a*); Khan *et al.*, (2005*b*); Nisar *et al.*, (2010); Riaz *et al.*, (2002); Sharif *et al.*, (2005).) of Pakistan. The plant *Dodonaea Viscosa* has been screened for the presence of biologically active compounds resulting in the isolation of a Flavonoid (Fig.1). The crystal structure and isolation of the title compound are presented below. Flavonoids, comprising a vast family of polyphenolic secondary metabolites, exhibit a wide range of biological activities, such as anti–oxidant (Pedrielli *et al.*, 2001), anti–viral (Lin *et al.*, 1999), anti–protozoal (Calzada, *et al.,*  1999).

The methoxy groups at C4 and C9 of title compound (Fig. 1) are nearly orthogonal to the benzopyranone moiety, as indicated by the torsion angles 97.5 (3)° and 107.6 (3)° respectively. The methoxy group at C15 is nearly coplanar with the phenyl ring with torsion angle (C17–C15–O7–C16) 0.3 (4)°. Rings *A* and *B* (the benzopyrone moiety) are fused at C1 and C7 and almost coplanar, the interplanar angle between the two rings is 1.40 (3)°. Ring *C* (the phenyl moeity) is attached to benzopyranone system at C11 with an interplanar angle of 5.83 (2)° between the two ring systems.

A combination of intermolecular and intramolecular hydrogen bonding, forming *R*^4^_6_(12) and *R*^4^_4_(16) patterns (Etter *et al.*, 1990), links the molecules into stepped ribbons perpendicular to *b* axis (Fig. 2 and Table 1). The ribbons are stacked parallel to the *b* axis by π–π interactions (Fig. 3); the average interplanar distance is 3.378 (3)Å (under symmetry operation 3/2-x, -1/2+y, 3/2-z) and the distance from the centroid of the phenyl group to the centre of the C1–C7 bond is 3.476 (3)Å.

### Experimental

The whole plant of *Dodonaea viscosa* (50 kg) was powdered and extracted with methanol (100 L × 3) at room temperature and the residue (1 kg) was separated under vacuum. The residue was suspended in water and extracted with *n*–hexane, chloroform, ethyl acetate and *n*–butanol respectively. The ethyl acetate fraction (250 g) was subjected repeatedly to column chromatography on silica gel using petroleum ether with a gradient of 25% chloroform to yield the title compound (50 mg). Single crystals suitable for X–ray diffraction analysis were obtained from an ether–chloroform mixture (1:2) by slow evaporation of the solvent at room temperature.

### Refinement

H atoms bonded to carbon and the phenol oxygen atoms were placed in geometric positions using a riding model, C—H distances were constrained as 0.95Å, 0.98Å and 0.84Å, for aryl, methyl and phenol groups respectively. Hydrogen atoms on the water molecule were located from difference maps and their coordinates refined under restraints. Thermal parameters were set to *U*_iso_(H) = 1.5*U*_eq_(C, O) for methyl groups and the water molecule and *U*_iso_(H) = 1.2*U*_eq_(C) for all others.

### Crystal data

**Table:**

C_18_H_16_O_7_·H_2_O	*F*(000) = 1520
*M**_r_* = 362.32	*D*_x_ = 1.456 Mg m^−^^3^
Monoclinic, *C*2/*c*	Mo *K*α radiation, λ = 0.71073 Å
Hall symbol: -C 2yc	Cell parameters from 1391 reflections
*a* = 19.869 (4) Å	θ = 2.6–22.0°
*b* = 6.8126 (15) Å	µ = 0.12 mm^−^^1^
*c* = 24.424 (5) Å	*T* = 150 K
β = 91.298 (4)°	Block, yellow
*V* = 3305.2 (12) Å^3^	0.19 × 0.18 × 0.09 mm
*Z* = 8	

### Data collection

**Table:**

Bruker APEXII CCD diffractometer	3400 independent reflections
Radiation source: fine–focus sealed tube	2072 reflections with *I* > 2σ(*I*)
graphite	*R*_int_ = 0.068
φ and ω scans	θ_max_ = 26.4°, θ_min_ = 1.7°
Absorption correction: multi-scan (*SADABS*; Sheldrick, 2008*a*)	*h* = −24→24
*T*_min_ = 0.978, *T*_max_ = 0.990	*k* = −8→8
14127 measured reflections	*l* = −30→30

### Refinement

**Table:**

Refinement on *F*^2^	Primary atom site location: structure-invariant direct methods
Least-squares matrix: full	Secondary atom site location: difference Fourier map
*R*[*F*^2^ > 2σ(*F*^2^)] = 0.050	Hydrogen site location: inferred from neighbouring sites
*wR*(*F*^2^) = 0.142	H atoms treated by a mixture of independent and constrained refinement
*S* = 1.05	*w* = 1/[σ^2^(*F*_o_^2^) + (0.0597*P*)^2^ + 1.2286*P*] where *P* = (*F*_o_^2^ + 2*F*_c_^2^)/3
3400 reflections	(Δ/σ)_max_ < 0.001
243 parameters	Δρ_max_ = 0.28 e Å^−^^3^
3 restraints	Δρ_min_ = −0.28 e Å^−^^3^

### Special details

**Table:**

Geometry. All s.u.'s (except the s.u. in the dihedral angle between two l.s. planes) are estimated using the full covariance matrix. The cell s.u.'s are taken into account individually in the estimation of s.u.'s in distances, angles and torsion angles; correlations between s.u.'s in cell parameters are only used when they are defined by crystal symmetry. An approximate (isotropic) treatment of cell s.u.'s is used for estimating s.u.'s involving l.s. planes.
Refinement. Refinement of *F*^2^ against ALL reflections. The weighted *R*–factor *wR* and goodness of fit *S* are based on *F*^2^, conventional *R*–factors *R* are based on *F*, with *F* set to zero for negative *F*^2^. The threshold expression of *F*^2^ > 2σ(*F*^2^) is used only for calculating *R*–factors(gt) etc. and is not relevant to the choice of reflections for refinement. *R*–factors based on *F*^2^ are statistically about twice as large as those based on *F*, and *R*–factors based on ALL data will be even larger.

### Fractional atomic coordinates and isotropic or equivalent isotropic displacement parameters (Å^2^)

**Table:**

	*x*	*y*	*z*	*U*_iso_*/*U*_eq_	
O1	0.69131 (8)	0.1125 (2)	0.73867 (6)	0.0233 (4)	
C1	0.65171 (12)	0.1096 (4)	0.69190 (10)	0.0229 (6)	
C2	0.58358 (12)	0.0898 (4)	0.69845 (10)	0.0258 (6)	
H2	0.5651	0.0824	0.7339	0.031*	
C3	0.54256 (13)	0.0807 (4)	0.65176 (10)	0.0263 (6)	
O2	0.47576 (9)	0.0548 (3)	0.65803 (8)	0.0367 (5)	
H2A	0.4573	0.0329	0.6274	0.063 (11)*	
C4	0.56984 (13)	0.0955 (4)	0.59938 (10)	0.0268 (6)	
O3	0.52807 (9)	0.0810 (2)	0.55366 (7)	0.0313 (5)	
C5	0.50843 (15)	0.2684 (4)	0.53157 (11)	0.0381 (7)	
H5A	0.4789	0.2488	0.4993	0.057*	
H5B	0.5486	0.3413	0.5210	0.057*	
H5C	0.4844	0.3429	0.5593	0.057*	
C6	0.63836 (13)	0.1158 (4)	0.59382 (10)	0.0248 (6)	
O4	0.66442 (10)	0.1283 (3)	0.54307 (7)	0.0326 (5)	
H4A	0.7066	0.1279	0.5456	0.079 (13)*	
C7	0.68135 (12)	0.1217 (4)	0.64058 (9)	0.0223 (5)	
C8	0.75264 (12)	0.1433 (4)	0.63673 (10)	0.0236 (6)	
O5	0.78120 (9)	0.1632 (3)	0.59130 (7)	0.0314 (5)	
C9	0.79061 (12)	0.1419 (4)	0.68777 (10)	0.0230 (6)	
O6	0.85888 (8)	0.1730 (3)	0.68523 (7)	0.0308 (5)	
C10	0.89618 (14)	0.0038 (5)	0.66731 (12)	0.0463 (8)	
H10A	0.9442	0.0360	0.6666	0.069*	
H10B	0.8805	−0.0341	0.6305	0.069*	
H10C	0.8892	−0.1054	0.6927	0.069*	
C11	0.75982 (12)	0.1256 (4)	0.73687 (10)	0.0227 (6)	
C12	0.79011 (12)	0.1236 (3)	0.79246 (10)	0.0216 (5)	
C13	0.85991 (12)	0.1219 (4)	0.80250 (10)	0.0264 (6)	
H13	0.8895	0.1229	0.7725	0.032*	
C14	0.88602 (13)	0.1188 (4)	0.85496 (10)	0.0253 (6)	
H14	0.9334	0.1153	0.8609	0.030*	
C15	0.84399 (12)	0.1208 (4)	0.89942 (10)	0.0225 (5)	
O7	0.87539 (8)	0.1210 (3)	0.94962 (7)	0.0300 (4)	
C16	0.83423 (14)	0.1220 (5)	0.99661 (10)	0.0407 (7)	
H16A	0.8629	0.1224	1.0298	0.061*	
H16B	0.8057	0.0047	0.9963	0.061*	
H16C	0.8058	0.2396	0.9960	0.061*	
C17	0.77463 (12)	0.1225 (4)	0.89071 (10)	0.0252 (6)	
H17	0.7453	0.1239	0.9209	0.030*	
C18	0.74882 (12)	0.1220 (4)	0.83780 (10)	0.0232 (6)	
H18	0.7014	0.1206	0.8321	0.028*	
O1W	0.88745 (11)	0.4465 (4)	0.57732 (8)	0.0492 (6)	
H1A	0.8602 (15)	0.357 (4)	0.5866 (12)	0.074*	
H1B	0.8908 (17)	0.435 (5)	0.5425 (7)	0.074*	

### Atomic displacement parameters (Å^2^)

**Table:**

	*U*^11^	*U*^22^	*U*^33^	*U*^12^	*U*^13^	*U*^23^
O1	0.0190 (9)	0.0306 (10)	0.0200 (9)	−0.0011 (7)	−0.0033 (7)	−0.0012 (8)
C1	0.0253 (13)	0.0201 (13)	0.0230 (13)	0.0015 (10)	−0.0049 (11)	−0.0001 (11)
C2	0.0245 (14)	0.0287 (15)	0.0243 (13)	0.0006 (11)	0.0005 (11)	−0.0022 (11)
C3	0.0240 (14)	0.0253 (15)	0.0294 (14)	−0.0005 (11)	−0.0028 (11)	−0.0007 (11)
O2	0.0221 (10)	0.0538 (14)	0.0340 (11)	−0.0035 (9)	−0.0051 (8)	−0.0030 (9)
C4	0.0340 (15)	0.0214 (14)	0.0246 (14)	0.0005 (11)	−0.0076 (11)	−0.0029 (11)
O3	0.0352 (11)	0.0284 (10)	0.0297 (10)	−0.0006 (8)	−0.0143 (8)	−0.0011 (8)
C5	0.0397 (17)	0.0334 (17)	0.0405 (17)	0.0053 (13)	−0.0154 (13)	0.0020 (13)
C6	0.0314 (15)	0.0206 (13)	0.0223 (13)	0.0029 (11)	−0.0003 (11)	−0.0013 (11)
O4	0.0339 (12)	0.0423 (12)	0.0217 (10)	0.0024 (9)	−0.0011 (8)	−0.0005 (8)
C7	0.0260 (13)	0.0190 (13)	0.0219 (13)	0.0025 (11)	−0.0018 (10)	−0.0010 (10)
C8	0.0277 (14)	0.0206 (13)	0.0226 (13)	0.0014 (11)	0.0029 (11)	−0.0005 (10)
O5	0.0288 (10)	0.0439 (12)	0.0217 (10)	0.0000 (9)	0.0026 (8)	0.0000 (8)
C9	0.0191 (13)	0.0246 (14)	0.0253 (13)	−0.0010 (10)	0.0012 (10)	−0.0005 (11)
O6	0.0211 (9)	0.0428 (12)	0.0285 (10)	−0.0036 (8)	0.0024 (8)	0.0014 (8)
C10	0.0263 (16)	0.072 (2)	0.0404 (17)	0.0160 (15)	0.0009 (13)	−0.0118 (16)
C11	0.0189 (13)	0.0216 (13)	0.0276 (14)	0.0014 (10)	−0.0004 (11)	−0.0009 (11)
C12	0.0229 (13)	0.0181 (13)	0.0238 (13)	−0.0001 (10)	−0.0028 (10)	−0.0014 (10)
C13	0.0236 (13)	0.0291 (14)	0.0267 (14)	−0.0003 (11)	0.0013 (11)	−0.0012 (12)
C14	0.0216 (13)	0.0264 (14)	0.0278 (14)	0.0005 (11)	−0.0027 (11)	0.0016 (11)
C15	0.0245 (13)	0.0189 (13)	0.0239 (13)	−0.0004 (10)	−0.0027 (11)	−0.0003 (10)
O7	0.0262 (10)	0.0426 (11)	0.0211 (9)	0.0009 (8)	−0.0041 (7)	0.0002 (8)
C16	0.0323 (16)	0.068 (2)	0.0211 (14)	−0.0006 (15)	−0.0012 (12)	0.0013 (14)
C17	0.0249 (14)	0.0281 (14)	0.0228 (13)	0.0010 (11)	0.0016 (11)	−0.0008 (11)
C18	0.0207 (13)	0.0242 (13)	0.0246 (13)	−0.0001 (11)	−0.0005 (10)	−0.0011 (11)
O1W	0.0424 (13)	0.0714 (17)	0.0334 (12)	−0.0225 (11)	−0.0046 (10)	0.0037 (11)

### Geometric parameters (Å, °)

**Table:**

O1—C11	1.366 (3)	O6—C10	1.444 (3)
O1—C1	1.372 (3)	C10—H10A	0.9800
C1—C2	1.373 (3)	C10—H10B	0.9800
C1—C7	1.399 (3)	C10—H10C	0.9800
C2—C3	1.388 (3)	C11—C12	1.473 (3)
C2—H2	0.9500	C12—C18	1.393 (3)
C3—O2	1.351 (3)	C12—C13	1.403 (3)
C3—C4	1.404 (4)	C13—C14	1.372 (3)
O2—H2A	0.8400	C13—H13	0.9500
C4—C6	1.378 (4)	C14—C15	1.385 (3)
C4—O3	1.380 (3)	C14—H14	0.9500
O3—C5	1.436 (3)	C15—O7	1.363 (3)
C5—H5A	0.9800	C15—C17	1.390 (3)
C5—H5B	0.9800	O7—C16	1.424 (3)
C5—H5C	0.9800	C16—H16A	0.9800
C6—O4	1.357 (3)	C16—H16B	0.9800
C6—C7	1.411 (3)	C16—H16C	0.9800
O4—H4A	0.8400	C17—C18	1.379 (3)
C7—C8	1.429 (3)	C17—H17	0.9500
C8—O5	1.265 (3)	C18—H18	0.9500
C8—C9	1.442 (3)	O1W—H1A	0.852 (17)
C9—C11	1.363 (3)	O1W—H1B	0.859 (17)
C9—O6	1.376 (3)		
			
C11—O1—C1	121.81 (19)	O6—C10—H10A	109.5
O1—C1—C2	116.9 (2)	O6—C10—H10B	109.5
O1—C1—C7	120.0 (2)	H10A—C10—H10B	109.5
C2—C1—C7	123.1 (2)	O6—C10—H10C	109.5
C1—C2—C3	118.1 (2)	H10A—C10—H10C	109.5
C1—C2—H2	121.0	H10B—C10—H10C	109.5
C3—C2—H2	121.0	C9—C11—O1	120.1 (2)
O2—C3—C2	118.2 (2)	C9—C11—C12	129.0 (2)
O2—C3—C4	120.9 (2)	O1—C11—C12	110.9 (2)
C2—C3—C4	120.9 (2)	C18—C12—C13	117.3 (2)
C3—O2—H2A	109.5	C18—C12—C11	119.8 (2)
C6—C4—O3	120.3 (2)	C13—C12—C11	122.9 (2)
C6—C4—C3	120.0 (2)	C14—C13—C12	121.0 (2)
O3—C4—C3	119.7 (2)	C14—C13—H13	119.5
C4—O3—C5	113.20 (19)	C12—C13—H13	119.5
O3—C5—H5A	109.5	C13—C14—C15	120.7 (2)
O3—C5—H5B	109.5	C13—C14—H14	119.7
H5A—C5—H5B	109.5	C15—C14—H14	119.7
O3—C5—H5C	109.5	O7—C15—C14	115.7 (2)
H5A—C5—H5C	109.5	O7—C15—C17	124.7 (2)
H5B—C5—H5C	109.5	C14—C15—C17	119.6 (2)
O4—C6—C4	119.6 (2)	C15—O7—C16	117.7 (2)
O4—C6—C7	120.1 (2)	O7—C16—H16A	109.5
C4—C6—C7	120.3 (2)	O7—C16—H16B	109.5
C6—O4—H4A	109.5	H16A—C16—H16B	109.5
C1—C7—C6	117.6 (2)	O7—C16—H16C	109.5
C1—C7—C8	120.2 (2)	H16A—C16—H16C	109.5
C6—C7—C8	122.2 (2)	H16B—C16—H16C	109.5
O5—C8—C7	122.3 (2)	C18—C17—C15	119.3 (2)
O5—C8—C9	121.5 (2)	C18—C17—H17	120.3
C7—C8—C9	116.2 (2)	C15—C17—H17	120.3
C11—C9—O6	121.0 (2)	C17—C18—C12	122.1 (2)
C11—C9—C8	121.6 (2)	C17—C18—H18	118.9
O6—C9—C8	117.2 (2)	C12—C18—H18	118.9
C9—O6—C10	113.8 (2)	H1A—O1W—H1B	105 (2)

### Hydrogen-bond geometry (Å, °)

**Table:**

*D*—H···*A*	*D*—H	H···*A*	*D*···*A*	*D*—H···*A*
O2—H2A···O1W^i^	0.84	1.92	2.712 (3)	157
O2—H2A···O3	0.84	2.33	2.780 (3)	114
O4—H4A···O5	0.84	1.85	2.589 (3)	146
O1W—H1A···O5	0.85 (2)	2.05 (2)	2.886 (3)	165 (3)
O1W—H1A···O6	0.85 (2)	2.71 (3)	3.288 (3)	126 (3)
O1W—H1B···O4^ii^	0.86 (2)	2.38 (2)	3.135 (3)	148 (3)

Symmetry codes: (i) *x*−1/2, *y*−1/2, *z*; (ii) −*x*+3/2, −*y*+1/2, −*z*+1.
